# Condylar morphology and position changes after miniscrew-assisted rapid palatal expansion in skeletal Class III malocclusion adult patients with mandibular deviation and unilateral posterior crossbite

**DOI:** 10.1186/s40510-022-00425-4

**Published:** 2022-09-01

**Authors:** Shuai Chen, Chunxi Zhang, Kaili Zhang, Xiaoming Tan, Xun Xi, Yi Zhao, Dongxu Liu

**Affiliations:** 1 Department of Orthodontics, School and Hospital of Stomatology, Cheeloo College of Medicine, Shandong University & Shandong Key Laboratory of Oral Tissue Regeneration & Shandong Engineering Laboratory for Dental Materials and Oral Tissue Regeneration, Jinan, 250012 China; 2 The Center of Stomatology, Qingdao Municipal Hospital Affiliated to Qingdao University, #5 Donghai Middle Road, Qingdao, 266000 China; 3 Department of Stomatology, Linyi Third People’s Hospital, Linyi, 276023 China

**Keywords:** Condyle, Miniscrew-assisted rapid palatal expansion, Adult, Cone-beam computed tomography, Mandibular deviation

## Abstract

**Background:**

To evaluate the morphological and positional changes of mandibular condyle after miniscrew-assisted rapid palatal expansion (MARPE) in skeletal Class III malocclusion adult patients with horizontal mandibular deviation (MD).

**Methods:**

The sample consisted of 15 patients with MD (6 males and 9 females, mean age 21.58 ± 3.12 years). The CBCT scans were taken before and after MARPE immediately. The pre- and post-registered images of the cranial base and mandible were measured, respectively, by Mimics.

**Results:**

After expansion, the distance between superior condylar point and the Frankfort horizontal plane on the deviated side and the non-deviated side increased by 0.96 ± 0.60 mm (*P* = 0.011) and 0.70 ± 0.65 mm (*P* = 0.046); coronal condylar angle of the deviated side increased by 0.39° ± 0.34 (*P* = 0.028) and 0.06° ± 0.49 (*P* = 0.917) on the non-deviated side. No statistically significant differences were found when comparing the condylar position on both sides before and after treatment. The degree of mandibular deviation decreased 0.43 mm (*P* = 0.270).

**Conclusions:**

This study suggested that for skeletal Class III malocclusion adult patients with horizontal MD, the condyle on the deviated side rotated toward the non-deviated side in the coronal direction; the condylar remodeling occurred mainly on the deviated side after MARPE in the vertical direction.

## Background

Skeletal Class III malocclusion is one of the most pervasive skeletal problems. It may be due to a retrognathic maxilla, a prognathic mandible, or both [1]. Skeletal Class III malocclusions are often accompanied by maxillary transverse deficiency (MTD) [2]. In addition, MTD has a 40% incidence rate of horizontal mandibular deviation (MD) [3], which is often manifested as deviated side crossbite maxillary ipsilateral molars with buccal inclination, mandibular molars with lingual inclination, facial asymmetry, dental midline discrepancy and chin deviation. Previous studies have shown that skeletal Class III malocclusion patients often have occlusal interference due to unilateral posterior crossbite, which forces the mandible to functionally shift to one side in order to establish a more stable occlusion, resulting in MD [4]. Therefore, MTD may be one of the causes of MD.

To solve the transverse problem, severe cases require orthodontic-orthognathic surgery [5]; mild to moderate discrepancies can be treated with orthodontic camouflage therapy [6]. For adolescents and adults with growth arrest, miniscrew-assisted rapid palatal expansion (MARPE) has become an important alternative to surgery to solve the MTD problem [7]. MAPRE is a unique type of implant-assisted maxillary expander, achieved by inserting four implants through the double cortical bone of the hard palate and the palatal bone to produce orthopedic force [8], ensuring the parallel expansion of the midpalatal suture [9]. Previous studies have focused on evaluating the dentoalveolar effects of MARPE, however, the condylar response to this procedure is not well understood, especially in patients with MD. It has been clinically reported that early intervention can promote condylar growth, correct mandibular deviation, achieving symmetrical growth of jaw bones [10–12]. However, the adaptive changes of the condyle in adults with Class III malocclusion and horizontal MD after expansion treatment remain to be evaluated.

The condyle is the growth center of the mandible, which is adapted to the surrounding environment through continuous structural reconstruction within a certain range. It has been found that the growth of the condyle increases in patients with Class II malocclusions after treatment with functional appliances such as the activator, Twin-block and Herbst [10, 13, 14]. For patients with growth and development, Arat et al. [11] and Torres et al. [12] observed that rapid maxillary expansion (RME) induced bone modeling in the condyle. Furthermore, based on handwrist radiography, the Herbst appliance has been shown to promote remodeling at the posterosuperior border in patients with minimal or no residual growth [15], and skeletal facial growth potential remained even after the age of 20 [16].

Cone-beam computed tomography (CBCT) has been shown to be accurate and reliable in evaluation of mandibular condyle position [17, 18]. After segment and 3D model reconstruction, the CBCT data could be superimposed to evaluate changes in the position of the upper airway, teeth and alveolar bone after orthodontic treatment [19–21]. This study aims to retrospectively and quantitatively evaluate the changes of the condylar position in skeletal Class III adult patients with horizontal MD after MARPE treatment by CBCT registration.

## Materials and methods

### Study design

The study was reviewed and approved by the Research Ethics Board of School of Stomatology (protocol number 20200802). The sample size was calculated based on an α of 0.05 and a β of 0.2 to achieve the power of 80% and to detect the difference of 1.56 mm in condylar height measurements between groups, with a 1.65 mm estimated standard deviation [22]. The power analysis indicated a sample size of 11 was required. Fifteen patients who received MARPE treatment were retrospectively selected from the CBCT database. The inclusion criteria were as follows: (1) the distance between Menton and the median sagittal plane was greater than 2 mm; (2) mandibular body asymmetry due to the bodily shift of the mandible to the deviated side [3]; (3) ANB < 0º; (4) no history of oral and maxillofacial trauma, surgery or orthodontic treatment; (5) permanent dentition; (6) aged 18–30 years; (7) the palatal sutures were successfully separated after treatment. Patients with craniofacial syndrome, systemic disease, and temporomandibular joint disease were excluded.

The baseline of the fifteen patients was as follows: 6 males and 9 females, mean age 21.58 ± 3.12 years (minimum age 18, maximum age 26), BMI 21.17 ± 1.83 kg/m^2^; the maxillary transverse deficiency was 0.91 ± 1.16 mm diagnosed by Pennsylvania method [23]. The Mentons were 8.37 ± 3.61 mm deviated horizontally to the median sagittal plane.

### Miniscrew-assisted rapid palatal expansion

**Fig.1:**
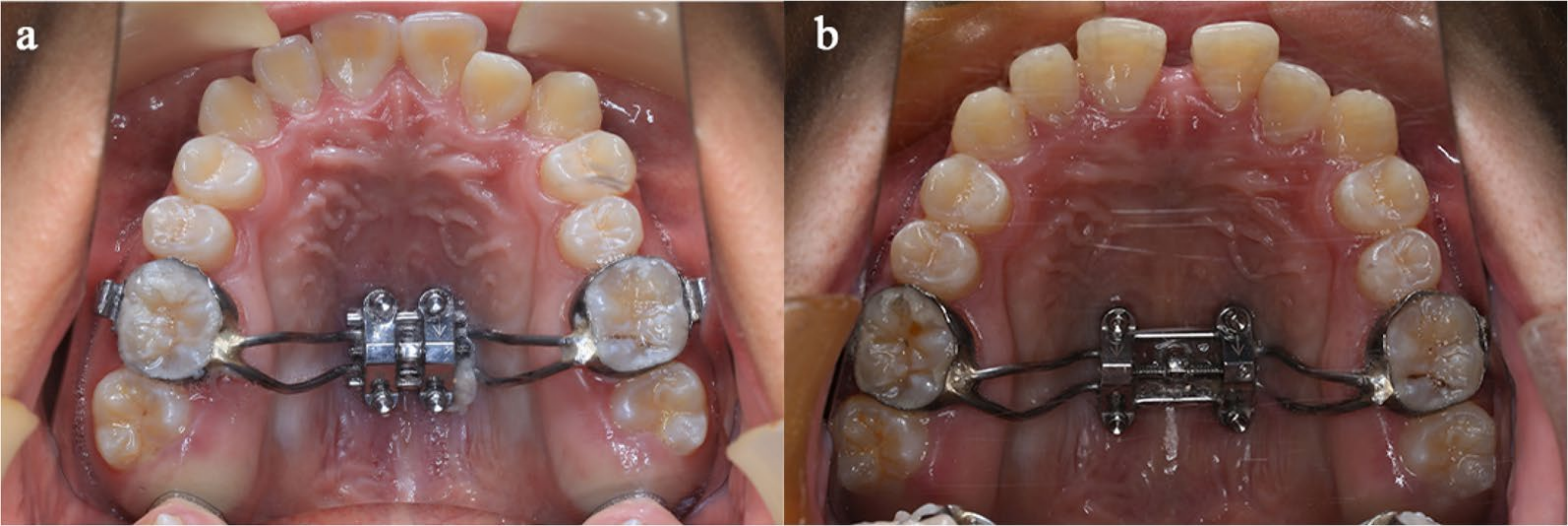
Intraoral view of MARPE. **a** before expansion; **b** after expansion

Patients were treated by the maxillary skeletal expansion appliance type-II (BioMaterials, Korea) under the supervision of the same clinician, which expanded by 0.8 mm in 6 turns. (Fig. 1). The appliance consisted of bands to the permanent first molars and four holes for mini-implants. To fenestrate the palatal base and nasal base, the matching orthodontic mini-implants (BioMaterials, Korea) are 1.8 mm in diameter and 11 mm in length. After 24 h of bonding with glass ionomer, the expander was activated one sixth of a turn (0.13 mm) in the morning and evening, respectively, until the occlusal aspect of the palatal cusp of the maxillary first molars contacted the occlusal aspect of the buccal cusp of the mandibular first molars. The duration of expansion was 18 ± 4.65 days.

### CBCT imaging

The CBCT scans were taken before (T1) and after MARPE treatment immediately (T2). All CBCT scans were implemented with each patient awake in the position of the Frankfort horizontal plane parallel to the floor using the same CBCT scanner (NewTom 5G, QR srl, Verona, Italy.) by the same operator. Patients were guided to close their mouths with the maximum intercuspation and the upper and lower lips and tongue were relaxed. The scanning range is from the frontal to the lower margin of the fourth cervical spine (standard voxel size: 0.3 mm; scan time: 14 s; slice thickness: 0.3 mm, 110 kV, 5 mA,). Subsequently, the dataset was exported in digital imaging and communications in medicine (DICOM) file format.

### Image registration

All the CBCT images were transferred into Materialse's interactive medical image control system (MIMICS, version 21.0; Materialise, Leuven, Belgium). Primarily, the head position of CBCT data was adjusted. In the axial view, the head position is rotated so that the sagittal axis passes through both the anterior nasal spine (ANS) and bason (Ba). In the coronal view, the head position is rotated so that the horizontal reference line is tangent to the bilateral orbitals (Or). Use the “along plane” command to make the Frankfort horizontal plane (passing through bilateral Or and right porion) parallel to the true horizontal plane. Secondly, thresholding based on Hounsfield Units was used to create the original cranial base mask (401HU-2347HU), maxillary mask (401HU-2347HU), and mandibular mask (822HU-3071HU). Thirdly, 3D virtual models of cranial base, maxilla and mandible were reconstructed from their masks respectively. Then, the three-dimensional image models of T1 were exported as stereolithography (STL) and imported into T2 CBCT data. Finally, 3D cranial base and mandibular superimpositions of T1 and T2 data were done by point registration followed with STL registration. Point registration of the cranial base were done by placing several obvious landmark points on the cranial base, for example, the anterior clinoid process, midpoint of anterior margin of foramen magnum, and so on. Afterward, anterior cranial base area was selected for the STL registrations to improve accuracy, and the whole cranial moved with it. The minimal point distance filter was set as 0.10 mm [20]. Similarly, the registration of mandible is also completed by those two steps above (landmark points: bilateral mandibular foramina, mental trigone, and genial tubercle; STL registration area: mandibular symphysis) [24].

### 3D measurement

**Fig.2:**
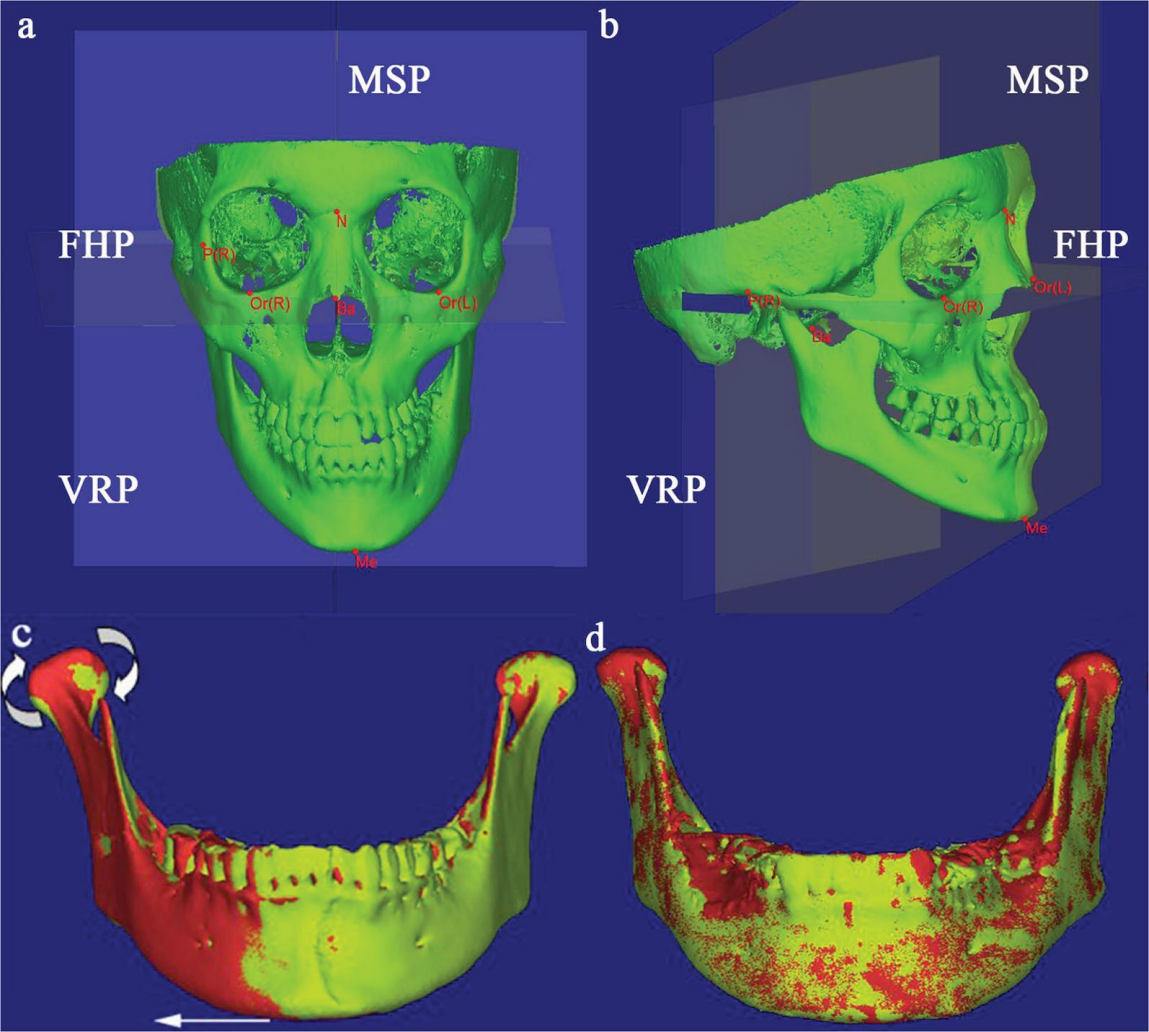
The deviated side of the mandible is on the patient's own left side. Three reference planes in **a** front view, **b** side view; 3D reconstruction model, the red one represents T2 and the yellow one represents T1: **c** changes of mandibular position after cranial base registration, **d** morphological changes after mandibular registration

As shown in Fig. 2a, b, the following three reference planes were established: (1) Frankfort horizontal plane (FHP) (2) Median sagittal plane (MSP): perpendicular to the FHP through basion (Ba) and nasion (N). (3) Vertical reference plane (VRP): passing through Ba and perpendicular to FHP and MSP. The landmarks and variables of measurement are shown in Tables 1 and 2. The one side with chin deviation was defined as the deviated side, whereas the other side was defined as the non-deviated side.

**Table 1:** Definition of landmarks

Landmarks	Definition
Superior condyle (SC)	The most superior point of the condylar head
Lateral condyle (LC)	The most lateral point of the condylar head
Medial condyle (MC)	The most medial point of the condylar head
Anterior condyle (AC)	The most anterior point of the condylar head
Posterior condyle (PC)	The most posterior point of the condylar head
Sigmoid notch (Sn)	The most inferior point on the sigmoid notch
Midpoint point of medial and lateral condyle (Mid-ML)	The midpoint of the line between MC and LC
Midpoint point of anterior and posterior condyle (Mid-AP)	The midpoint of the line between AC and PC
Condylar center (CC)	The midpoint of the line between Mid-ML and Mid-AP
Menton (Me)	The most inferior midpoint on the symphysis
Gonion (Go)	The most posterior inferior point on the outline of the angle of the mandible
Mesial palatal cusp (MPC)	The mesial palatal cusp of the maxillary first molar

**Table 2:** Definition of measurement variables

Measurement variables	Definition
Condylar distance (CC)	Distance between condyle centers
Axial condylar angle (ACA)	The angle between CC and MSP and VRP
Coronal condylar angle (CCA)	The angle between CC and MSP and FHP
Anteroposterior position of the condyle	The distance from condylar landmarks to VRP
Vertical position of the condyle	The distance from condylar landmarks to FHP
Lateral position of the condyle	The distance from condylar landmarks to MSP
Mandibular body length (MBL)	The distance between Go and Me
Mandibular ramus length (MRL)	The distance between SC and Go
Vertical position of the mesial palatal cusp	The distance from MPC to FHP

### Statistical analysis

Statistical analysis was performed by SPSS (version 20.0, IBM, New York, USA) software package. The intra-examiner reliability was determined by performing the measurements for each CBCT image on 2 separate occasions by one examiner at a 2-week interval. The intraclass correlation coefficients were calculated; then the mean of the 2 measurements was used in statistical analysis. The error of method was calculated using the Dahlberg formula:$$ME = \sqrt {{\raise0.7ex\hbox{${\Sigma (d)^{2} }$} \!\mathord{\left/ {\vphantom {{\Sigma (d)^{2} } {2n}}}\right.\kern-\nulldelimiterspace} \!\lower0.7ex\hbox{${2n}$}}}$$. For normal distribution data, paired t test was used to compare the difference between T1 and T2 for samples. In case of abnormal distribution, Wilcoxon signed rank test was used for comparison. *P* < 0.05 indicated that the difference was significant.

## Results

The intraclass correlation coefficients for all measurements ranged from 0.92 to 0.95, indicating sufficient reliability. The method error ranged from 0.14 mm to 0.21 mm for linear measurements and from 0.11° to 0.17° for angular measurement.

The midpalatal suture opened by 3.36 ± 0.69 mm at the maxillary first molar when comparing T2 to T1 CBCT data. The degree of mandibular deviation (Me-MSP) decreased 0.43 mm (*P* = 0.270), but the variation was not statistically significant (Fig. 2).

**Fig.3:**
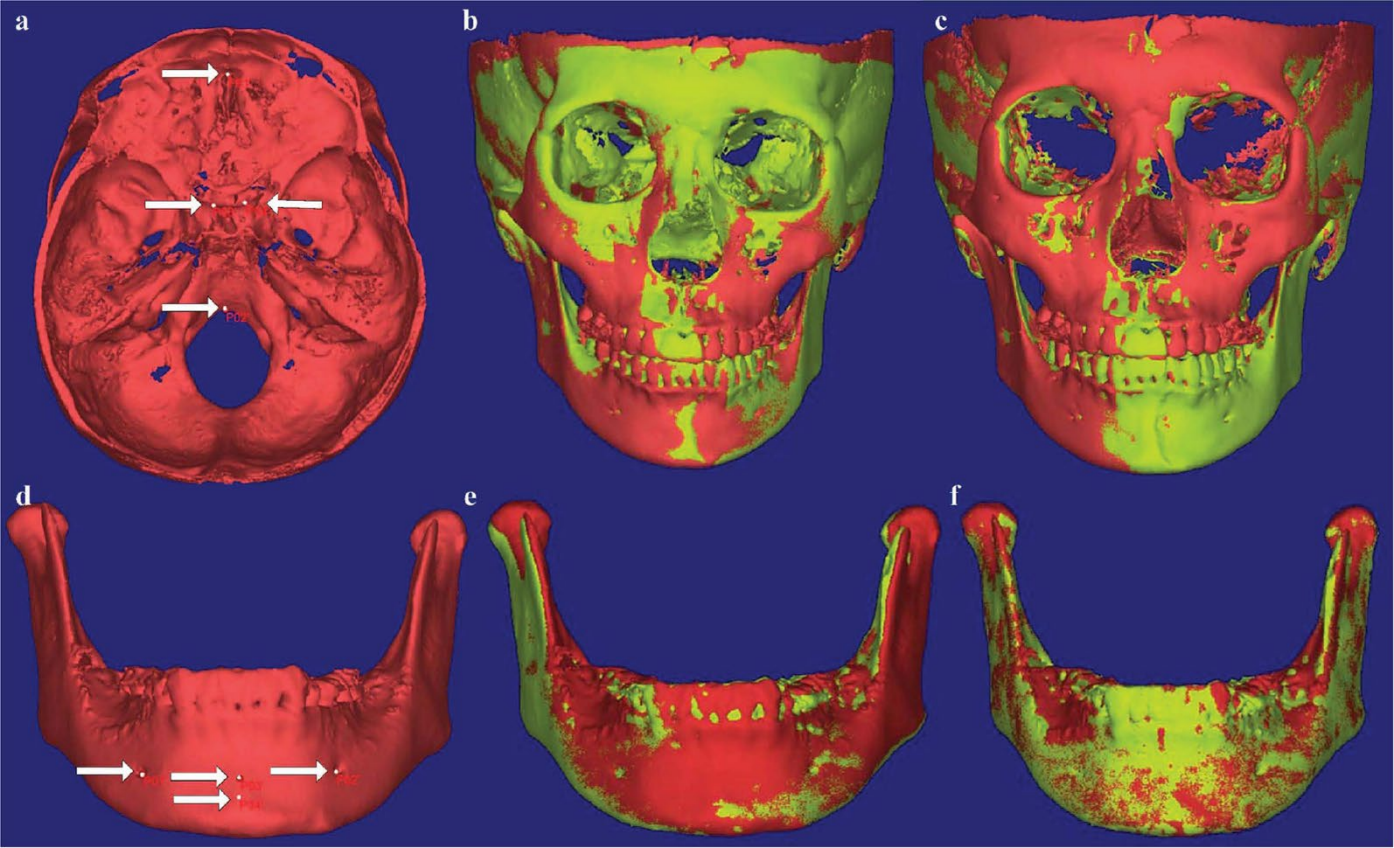
The deviated side of the mandible is on the patient's own left side. **a**, **d** landmark points of cranial base and mandibular superimpositions, respectively; **b**, **e** frontal view of point registration; **c**, **f** frontal view of STL registration

**Fig.4:**
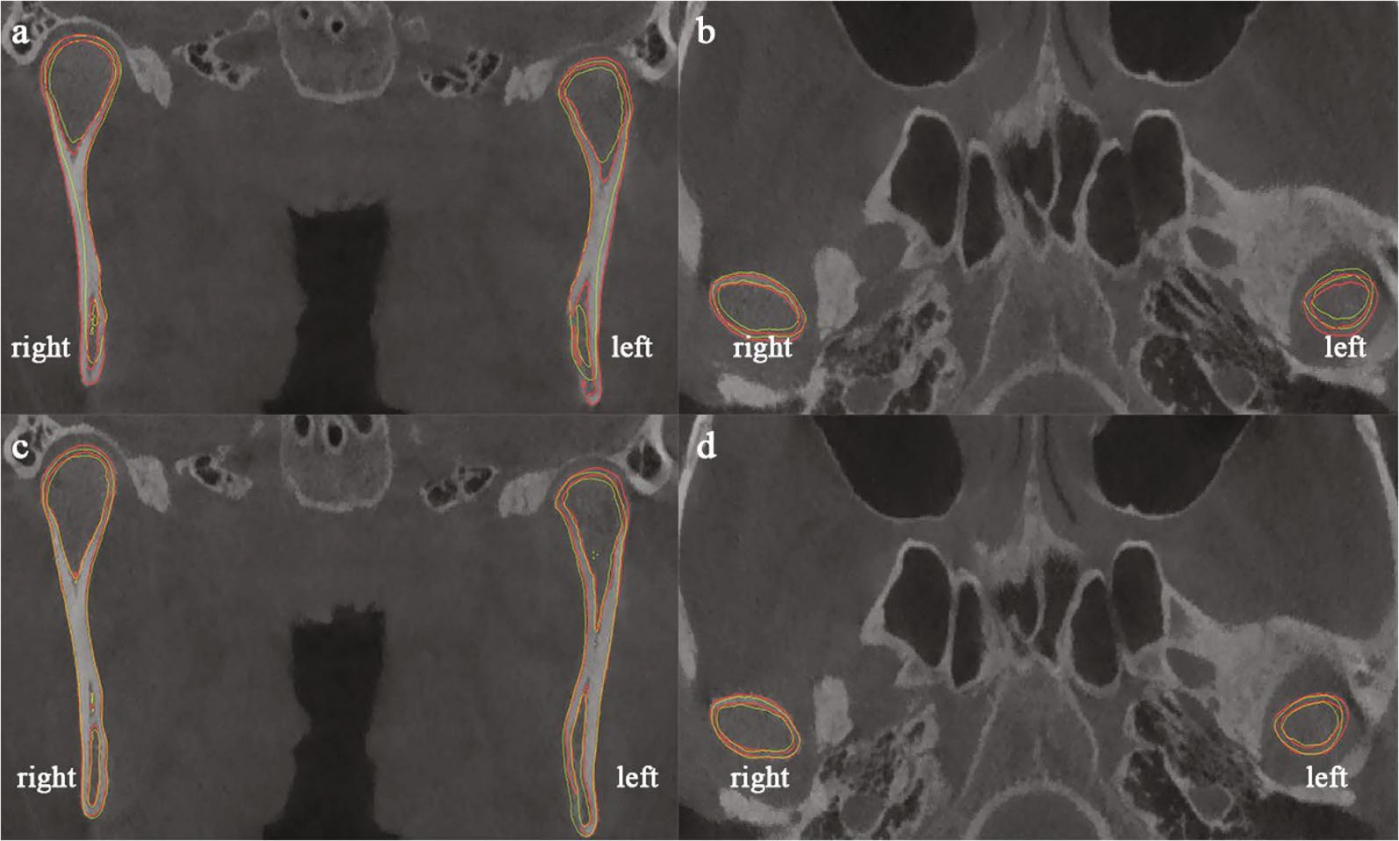
The deviated side of the mandible is on the patient's own left side. The red image represents T2 and the yellow image represents T1. **a**, **b**: cranial base registration; **c**, **d**: mandibular registration

According to the linear measurements (Table 3 and Fig. 3), the SC-MSP of the deviated side increased by 0.66 ± 0.61 mm, and that of the non-deviated side increased by 0.13 ± 0.53 mm, but only the former had statistical significance. There were no significant changes in other linear measurements of condylar position (Table 4). Considering the condylar changes in the vertical dimension, combined with the results of mandibular registration, the SC-FHP between the deviated side and the non-deviated side decreased by 0.96 ± 0.60 mm (*P* = 0.011) and 0.70 ± 0.65 mm (*P* = 0.046), respectively (Table 5 and Fig. 4). In terms of comparing the changes of condyles on both sides of mandibular deviation, the changes of partial condyle landmarks on both sides showed inconsistent trends based on cranial base registration (Table 6), while the changes of SC points on both sides showed consistent trends based on mandibular registration (Table 7).

**Table 3:** Comparison of condylar position and angle between deviated and non-deviated sides at T1 and T2, respectively (cranial base registration)

Variables	T1		Difference (DS-NDS)	*P*	T2		Difference (DS-NDS)	*P*
	DS	NDS			DS	NDS		
	Mean SD	Mean SD			Mean SD	Mean SD		
SC-MSP (mm)	49.87 0.95	49.67 2.96	0.21	0.859^†^	50.53 1.19	49.79 3.35	0.74	0.534^†^
SC-FHP (mm)	1.213 0.95	1.83 0.98	− 0.61	0.193^†^	0.68 0.87	1.33 0.84	- 0.65	0.127^†^
SC-VRP (mm)	13.05 1.88	13.98 2.61	- 0.93	0.470^†^	12.87 1.52	14.14 2.70	− 1.28	0.314^†^
AC-MSP (mm)	50.88 2.15	50.89 3.85	- 0.01	0.989^†^	50.71 2.40	50.88 3.87	− 0.17	0.867^†^
AC-FHP (mm)	4.98 2.42	5.70 2.45	- 0 .73	0.219^†^	5.04 2.49	5.39 2.56	− .36	0.462^†^
AC-VRP (mm)	16.52 1.79	18.46 2.55	- 1.94	0.186^†^	16.19 1.70	18.38 2.49	− 2.19	0.148^†^
PC-MSP (mm)	51.48 2.37	53.00 4.17	- 1.52	0.233^†^	51.55 2.64	53.04 4.20	− 1.49	0.204^†^
PC-FHP (mm)	6.01 1.65	6.46 1.82	- 0.45	0.249^‡^	5.91 1.72	6.25 1.75	− 0.33	0.463^‡^
PC-VRP (mm)	9.31 1.75	10.67 3.50	- 1.37	0.315^†^	8.73 1.07	10.57 3.61	− 1.84	0.236^†^
LC-MSP (mm)	59.55 3.00	60.15 4.50	- 0.60	0.599^†^	59.72 2.98	60.33 4.63	− 0.61	0.635^†^
LC-FHP (mm)	5.92 2.20	8.14 1.87	- 2.22	0.101^†^	6.83 2.48	6.42 2.47	0.40	0.776^†^
LC-VRP (mm)	15.89 2.73	16.74 4.87	- 0.85	0.345^‡^	15.70 2.39	16.95 5.02	− 1.25	0.428^†^
MC-MSP (mm)	42.32 0.41	43.26 3.35	- 0.94	0.508^†^	42.17 0.62	43.11 3.25	− 0.94	0.518^†^
MC-FHP (mm)	5.43 1.34	6.60 1.82	- 1.16	0.120^†^	5.27 1.47	6.48 2.29	− 1.21	0.085^†^
MC-VRP (mm)	12.57 2.10	13.34 2.01	- 0.77	0.583^†^	12.25 1.76	13.24 1.95	− 0.99	0.753^‡^
ACA (°)	75.27 1.70	74.11 2.67	1.16	0.314^†^	74.91 2.12	74.16 2.76	0.752	0.712^†^
CCA (°)	78.63 11.96	78.16 11.97	0.47	0.686^‡^	78.24 11.85	78.11 11.87	0.133	0.345^‡^
CC (mm)	102.88 5.52				103.11 5.61		− 0.235	0.151^†^

DS, deviated side; NDS, non-deviated side; Abbreviations are summarized in Table 1

^†^paired *t* test; ^‡^Wilcoxon signed rank test

**Table 4:** Comparison of condylar position and angle changes between deviated sides and non-deviated side sides at T1 and T2 (cranial base registration)

Variables	T1	T2	Difference (DS_T1_-DS_T2_)	P	T1	T2	Difference (NDS_T1_-NDS_T2_)	P
	DS	DS			NDS	NDS		
	Mean SD	Mean SD	Mean SD		Mean SD	Mean SD	Mean SD	
SC-MSP (mm)	49.87 0.95	50.53 1.19	− 0.66 0.61	0.045*^†^	49.67 2.96	49.79 3.35	− 0.13 0.53	0.581^†^
SC-FHP (mm)	1.21 0.95	0.68 0.87	0.53 0.34	0.012*^†^	1.83 0.98	1.33 0.84	0.50 0.22	0.002*^†^
SC-VRP (mm)	13.05 1.88	12.87 1.52	0.18 0.55	0.457^†^	13.98 2.61	14.14 2.70	− 0.16 0.19	0.437^†^
AC-MSP (mm)	50.88 2.15	50.71 2.40	0.17 0.65	0.552^†^	50.89 3.85	50.88 3.87	0.01 0.22	0.887^†^
AC-FHP (mm)	4.98 2.42	5.04 2.49	− 0.06 0.29	0.633^†^	5.70 2.45	5.39 2.56	0.31 0.35	0.083^†^
AC-VRP (mm)	16.52 1.79	16.19 1.70	0.33 0.60	0.235^†^	18.46 2.55	18.38 2.49	0.08 0.35	0.605^†^
PC-MSP (mm)	51.48 2.37	51.55 2.64	− 0.08 0.51	0.731^†^	53.00 4.17	53.04 4.20	− 0.05 0.22	0.631^†^
PC-FHP (mm)	6.01 1.65	5.91 1.72	0.10 0.35	0.500^‡^	6.46 1.82	6.25 1.75	0.22 0.35	0.192^†^
PC-VRP (mm)	9.31 1.75	8.73 1.07	0.58 0.74	0.115^†^	10.67 3.50	10.57 3.61	0.10 0.51	0.646^†^
LC-MSP (mm)	59.55 3.00	59.72 2.98	− 0.17 0.36	0.299^†^	60.15 4.50	60.33 4.63	− 0.18 0.25	0.140^†^
LC-FHP (mm)	5.92 2.20	6.83 2.48	− 0.91 2.63	0.437^†^	8.14 1.87	6.42 2.47	1.72 2.99	0.219^†^
LC-VRP (mm)	15.89 2.73	15.70 2.39	0.19 0.65	0.345^‡^	16.74 4.87	16.95 5.02	− 0.22 0.59	0.410^†^
MC-MSP (mm)	42.32 0.41	42.17 0.62	0.15 0.38	0.372^†^	43.26 3.35	43.11 3.25	0.15 0.33	0.312^†^
MC-FHP (mm)	5.43 1.34	5.27 1.47	0.16 0.64	0.567^†^	6.60 1.82	6.48 2.29	0.12 0.73	0.709^†^
MC-VRP (mm)	12.57 2.10	12.25 1.76	0.33 0.55	0.209^†^	13.34 2.01	13.24 1.95	0.10 0.74	0.463^‡^
ACA (°)	74.91 2.12	75.27 1.70	− 0.36 0.66	0.239^†^	74.16 2.76	74.11 2.67	0.05 0.37	0.773^†^
CCA (°)	78.24 11.85	78.63 11.96	− 0.39 0.34	0.028*^‡^	78.11 11.87	78.16 12.00	− 0.06 0.49	0.917^‡^

DS, deviated side; NDS, non-deviated side; *indicates a statistical significance at P < 0.05. ^†^paired *t* test; ^‡^Wilcoxon signed rank test

**Table 5:** Comparison of condylar position between deviated and non-deviated sides at T1 and T2, respectively (mandibular registration)

Variables	T1	T2	Difference (DS_T1_-DS_T2_)	*P*	T1	T2	Difference (NDS_T1_-NDS_T2_)	*P*
	DS	DS			NDS	NDS		
	Mean SD	Mean SD	Mean SD		|Mean SD|	|Mean SD|	Mean SD	
SC-MSP(mm)	50.64 1.00	50.52 1.20	0.12 0.88	0.753^†^	49.85 3.43	49.79 3.35	0.06 2.78	0.961^†^
SC-FHP(mm)	1.62 1.03	0.66 0.75	0.96 0.60	0.011*^†^	2.11 0.80	1.41 0.74	0.70 0.65	0.046*^†^
SC-VRP(mm)	12.40 1.86	12.82 1.67	− 0.42 2.49	0.698^†^	13.78 2.60	14.11 2.75	− 0.33 2.34	0.742^†^

DS, deviated side; NDS, non-deviated side; *indicates a statistical significance at *P* < 0.05. Abbreviations are summarized in Table 1

^†^paired *t* test

**Table 6:** Comparison of the difference between T1-T2 on the deviated and non-deviated sides (cranial base registration)

Variables	DS_T1_− DS_T2_	NDS_T1_− NDS_T2_	*P*
	Mean SD	Mean SD	
SC− MSP (mm)	− 0.66 0.61	− 0.13 0.53	0.394‡
SC− FHP (mm)	0.53 0.34	0.50 0.22	0.835†
SC− VRP (mm)	0.18 0.55	− 0.16 0.19	0.442†
AC− MSP (mm)	0.17 0.65	0.01 0.22	0.658†
AC− FHP (mm)	− 0.06 0.29	0.31 0.35	0.180‡
AC− VRP (mm)	0.33 0.60	0.08 0.35	0.302†
PC− MSP (mm)	− 0.08 0.51	− 0.05 0.22	1.000‡
PC− FHP (mm)	0.10 0.35	0.22 0.35	0.636†
PC− VRP (mm)	0.58 0.74	0.10 0.51	0.249†
LC− MSP (mm)	− 0.17 0.36	− 0.18 0.25	0.961†
LC− FHP (mm)	− 0.91 2.63	1.72 2.99	0.093‡
LC− VRP (mm)	0.19 0.65	− 0.22 0.59	0.193†
MC− MSP (mm)	0.15 0.38	0.15 0.33	0.990†
MC− FHP (mm)	0.16 0.64	0.12 0.73	0.906†
MC− VRP (mm)	0.33 0.55	0.10 0.74	0.310‡

DS, deviated side; NDS, non-deviated side; †paired t test; ‡Wilcoxon signed rank test

**Table 7:** Comparison of the difference between T1-T2 on the deviated and non-deviated sides (mandibular registration)

Variables	DS_T1_-DS_T2_	NDS_T1_-NDS_T2_	**P**
	Mean SD	Mean SD	
SC-MSP(mm)	0.12 0.88	0.06 2.78	0.957^†^
SC-FHP(mm)	0.96 0.60	0.70 0.65	0.503^†^
SC-VRP(mm)	− 0.42 2.49	− 0.33 2.34	0.926^†^

DS, deviated side; NDS, non-deviated side; †paired *t* test

**Fig.5:**
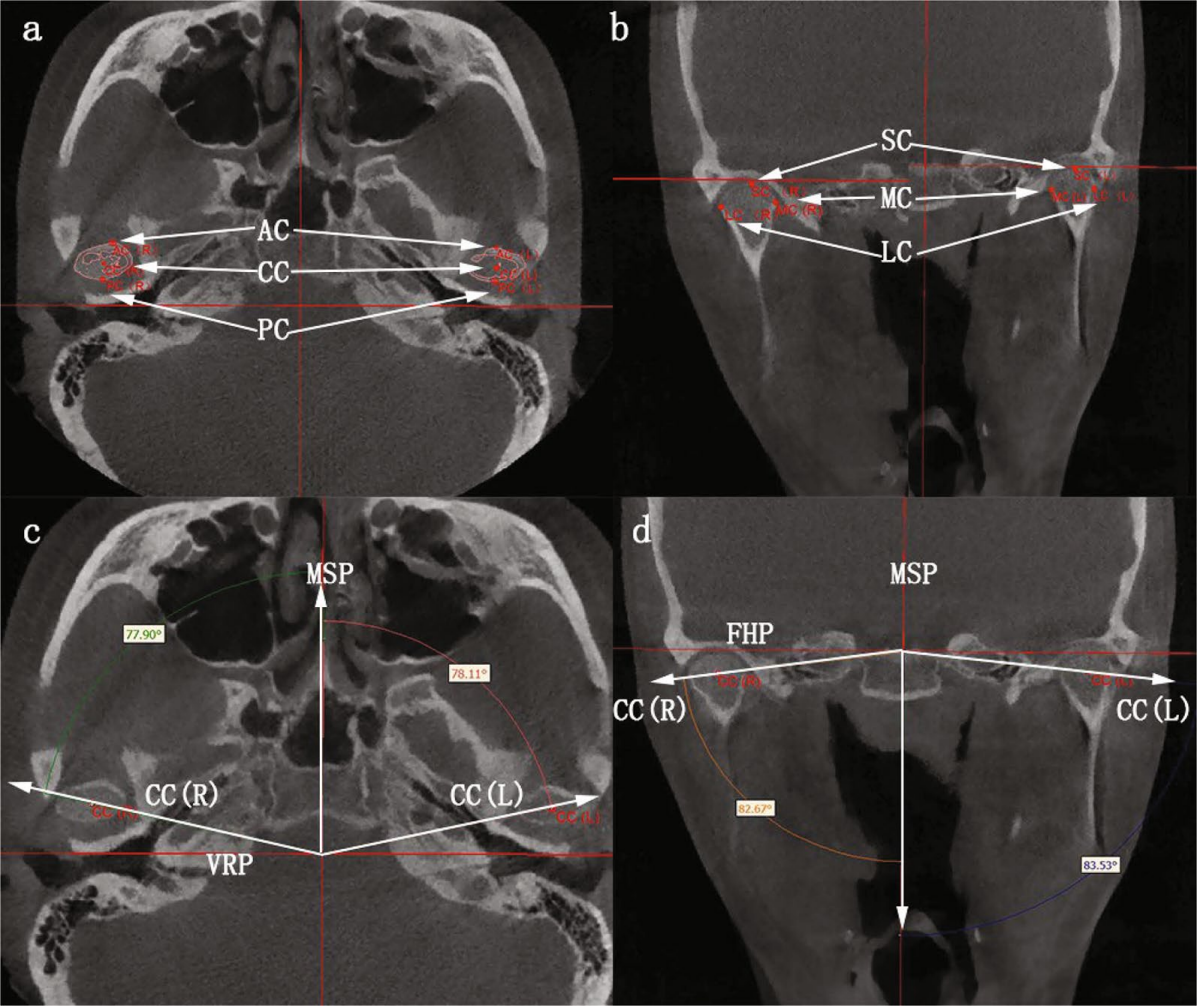
**a**, **b** condylar landmarks; **c** Axial condylar angle; **d** Coronal condylar angle

The axial condylar angle (ACA) of the deviated side increased by (0.36 ± 0.66)°, and the non-deviated side decreased by (0.05 ± 0.37)°, with no statistical significance. Horizontally, the condyle of the deviated side rotated medially, while the non-deviated side rotated laterally. The coronal condylar angle (CCA) of the deviated side increased by (0.39 ± 0.34)° after treatment, and there was no significant difference in the non-deviated side (Table 4 and Fig. 5).

The MPC-FHP on the deviated side increased by 0.27 ± 0.27 mm, while the contralateral side increased by 0.34 ± 0.24 mm. Before expansion, the mandibular body length (MBL) and mandibular ramus length (MRL) on the non-deviated side were 3.30 ± 1.69 mm (*P* = 0.012) and 3.05 ± 2.93 mm (*P* = 0.081) larger than those of the deviated side, respectively. After expansion, the difference of the latter was 2.92 ± 3.64 mm (*P* = 0.147).

## Discussion

This study found that the condyle was re-established in young adult patients after MARPE, which may be related to the remodeling of the condyle and the rotation of the mandible. In other words, the improvement of maxilla-mandible transverse relationship could improve MD to some extent. The result is consistent with that of the RME research in developing patients [25–27].

On the other hand, CCA significantly increased by 0.39 ± 0.34° on the deviated side and 0.06 ± 0.49° on the non-deviated side after expansion. It is consistent with the results of Melgaco et al. [28], but the amount of change is much smaller. The results may be due to the greater degree of modeling on the deviated side than on the opposite side, resulting in more upward movement of the condylar center. Combined with the significant increase in SC-MSP on the deviated side, the results indicated a tendency of lateral inclination of the condyles. At the same time, the lateral of the condyle on the deviated side moved downward, while the non-deviated side was opposite, and the medial side of the deviated side moved upward relative to the lateral side, while the non-deviated side was opposite, indicating that the condylar deviated side tended to rotate toward the non-deviated side in the coronal direction. On the basis of axial or coronal images and with reference to the median sagittal plane, the superior, posterior, and lateral condyle on the deviated side and the non-deviated side moved laterally, and the anterior and medial condyle moved medially. In general, the displacement of the deviated side was greater than that of the non-deviated side after MARPE, which could also explain the above rotation that helps improve mandibular deviation. Melgaco et al. [28] believed that occlusal changes and muscle extension caused by maxillary expansion will change the stress and its distribution of the mandible and condyle, which may lead to condylar rotation. However, the average reduction of Me-MSP was only 0.43 mm, which may be influenced by the excessive MBL on the non-deviated side, resulting in insignificant improvement in mandibular deviation.

Condylar bone modeling occurs throughout the whole life, with the changes of mechanical balance [29]. Sato et al. [30] found that the condyle cartilage thickness on the non-deviated side increased after the lateral deviation of mandible in rats, however, the cartilage thickness and labeling index of the condyle on the deviated side increased to a degree similar to the normal control group after the induction was removed, indicating that the condyle would model with occlusal changes [31]. Likewise, after using MARPE to solve the posterior crossbite, the occlusal interference was removed, which led to more condylar remodeling on the deviated side. In this study, the vertical height of the mesial palatal cusp of the maxillary first molar on both sides increased after expansion, which may force the mandible to move downward, causing the condyles on both sides to be subjected to tensile stress, thereby stimulating the corresponding remodeling of the condyles. Mongini et al. [32] believed that the modeling of bilateral condylar symmetry after orthodontic treatment resulted in the normalization of mandibular and condylar growth parameters, and compensatory mandibular and condylar growth mainly occurs on the deviated side of the mandible, which is consistent with the results of this study.

There are still some limitations in this study. First, the sample size of this study was quite limited. Larger sample size and a control group should be established to reach a higher level of evidence. Secondly, since adaptive changes in the TMJ occur over a long period of time, long-term follow-up is needed to determine the stability of condylar changes after MARPE treatment.

## Conclusions

- After MARPE, the condyle of the deviated side rotated to the non-deviated side, and the mandible moved downward and backward, which was helpful to improve the skeletal Class III relationship and mandibular deviation.
- Condylar remodeling was observed in both sides of Class III malocclusion adult patients with horizontal mandibular deviation, especially on the deviated side after MARPE.
- In Class III malocclusion adult patients with maxillary transverse deficiency and mild mandibular deviation, width correction may help to avoid surgery, thereby reducing costs and risks.

## Data Availability

The datasets used and/or analyzed during the current study are available from the corresponding author on reasonable request.

## Abbreviations

MARPE: Miniscrew-assisted rapid palatal expansion
MD: Mandibular deviation
CBCT: Cone-beam computed tomography
Mimics: Materialse's interactive medical image control system
MTD: Maxillary transverse deficiency
RME: Rapid maxillary expansion
DICOM: Digital imaging and communications in medicine
HU: Hounsfield units
STL: Stereolithography
